# Epiploic Appendagitis in Three Young Men: CT Diagnosis, Clinical Mimics, and Conservative Management

**DOI:** 10.7759/cureus.91895

**Published:** 2025-09-09

**Authors:** Usamah Al-Anbagi, Muayad K Ahmad, Mohamed Mohamedali, Abdulqadir J Nashwan, Hatem Abdulmajeed

**Affiliations:** 1 Internal Medicine Department, Hamad Medical Corporation, Doha, QAT; 2 Nursing and Midwifery Research Department, Hamad Medical Corporation, Doha, QAT; 3 Clinical Imaging Department, Hamad Medical Corporation, Doha, QAT

**Keywords:** acute abdominal pain, appendicitis, case series, computed tomography (ct), diverticulitis, epiploic appendagitis (ea)

## Abstract

Epiploic appendagitis (EA) is an uncommon, self-limiting inflammation of the small fat-filled sacs along the colon, occurring in approximately 0.3-1% of suspected appendicitis cases and 2-7% of suspected diverticulitis cases. EA can be primary (due to torsion or venous thrombosis) or secondary (associated with adjacent inflammatory conditions). We present a series of three male patients with primary EA, aged 25-37 years, who presented with sudden, localized abdominal pain variably involving the left lower quadrant, right lower quadrant, or flank. All were hemodynamically stable and afebrile; leukocyte counts and C-reactive protein were normal or only mildly elevated (C-reactive protein (CRP) up to 11 mg/L in two patients). Computed tomography (CT) scans revealed characteristic ovoid fat-density lesions with surrounding inflammatory stranding adjacent to the colon. Each patient was managed conservatively with paracetamol, fluids, and rest, avoiding unnecessary antibiotics, hospital admission, and surgery. Symptoms resolved within 7-10 days, with no recurrence at follow-up. These cases underscore the importance of recognizing EA to prevent overtreatment and highlight its variable presentation and favorable response to conservative management.

## Introduction

Epiploic appendagitis (EA) is a rare, benign, and self-limiting ischemic/infarction-type inflammatory condition involving the epiploic appendages - fat-filled serosal outpouchings that line the anti-mesenteric surface of the colon [1,2]. EA may be primary, caused by torsion or spontaneous thrombosis of the central draining vein, or secondary, occurring adjacent to inflammatory conditions, such as diverticulitis, appendicitis, or cholecystitis [3,4]. Its true incidence remains uncertain, but it is estimated to account for 0.3-1% of patients initially suspected to have appendicitis and 2-7% of those suspected to have diverticulitis [4]. Several risk factors have been described, including obesity, rapid weight loss, and vigorous physical activity [2].

Typical sites of EA include the left lower quadrant (most frequent) and right lower quadrant, reflecting the higher density of epiploic appendages in these regions [5,6]. Patients usually present with sudden, localized abdominal pain and minimal or absent systemic features such as fever or leukocytosis [3,5]. Importantly, unlike diverticulitis, EA is not associated with bowel wall thickening [6]. The main mimics of EA are acute appendicitis, acute diverticulitis, and omental infarction [3,6]. Less commonly, other conditions, such as hernia or complications after heavy meals, have been described, though these are not consistently supported in the literature. Because of this overlap, EA is often misdiagnosed, leading to unnecessary hospitalization, antibiotics, or even surgery.

Contrast-enhanced computed tomography (CT) is the gold standard for diagnosis, showing an ovoid fat-density lesion adjacent to the colon with surrounding inflammatory stranding but without bowel wall thickening [6-9]. Confirmation by a radiologist is essential. While abdominal ultrasound can sometimes be useful, especially in thin patients, it is less sensitive and operator-dependent. Management is conservative, consisting mainly of non-steroidal anti-inflammatory drugs (NSAIDs) for pain relief, with resolution of symptoms usually occurring within one to two weeks [2,10]. Surgical intervention is rarely required, except in cases of diagnostic uncertainty or complications.

This case series describes three young men diagnosed with EA by CT and confirmed by a radiologist. We highlight their clinical and radiological features, emphasize the diagnostic challenges posed by EA, and stress the importance of recognizing this underdiagnosed entity to avoid overtreatment.

## Case presentation

Case 1

**Figure 1:**
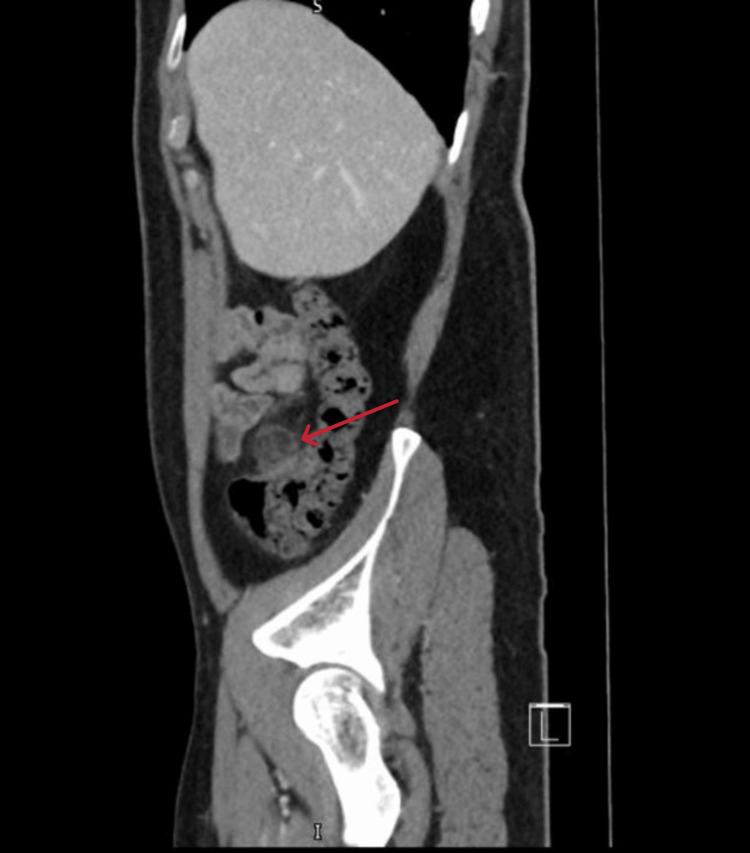
Coronal computed tomography image of epiploic appendagitis The image shows an ovoid fat-density lesion adjacent to the junction of the descending and sigmoid colon (red arrow), with surrounding inflammatory fat stranding and thickened peritoneum, consistent with epiploic appendagitis.

A 25-year-old male presented with a four-day history of constant, sharp left flank pain, 3-4/10 numeric rating scale (NRS), radiating to the left lower quadrant, not relieved by bowel movement or posture, and without urinary symptoms or hematuria, associated with watery diarrhea occurring four times daily. He denied fever, chills, or trauma. On examination, he was afebrile and hemodynamically stable, with a soft, non-tender abdomen and normal cardiopulmonary findings. Laboratory tests were within normal limits, including WBC of 7.2 × 10^9^/L, C-reactive protein (CRP) of 2 mg/L, and normal renal and electrolyte profiles (Table 1). A contrast-enhanced abdominal CT scan revealed an ovoid fat-density lesion with surrounding inflammatory stranding and a thin hyperattenuating rim surrounding the lesion near the junction of the descending and sigmoid colon, consistent with epiploic appendagitis (Figure 1). The patient was managed conservatively with paracetamol, fluids, and rest, without antibiotics or surgery. Symptoms were resolved within seven days, and the patient was doing well on follow-up.

**Table 1:** Laboratory results of the three presented cases with reference ranges WBC = white blood cell count; CRP = C-reactive protein; µmol/L = micromoles per liter; mmol/L = millimoles per liter; mg/L = milligrams per liter “Normal” indicates a result within the stated reference range for the reporting laboratory.

Parameter	Case 1	Case 2	Case 3	Reference Range
White blood cell count (× 10⁹/L)	7.2 × 10⁹/L	12.56 × 10⁹/L	10.2 × 10⁹/L	4.0-11.0 × 10⁹/L
Neutrophil %	60%	73%	69%	40–70%
C-reactive protein (mg/L)	2 mg/L	11 mg/L	11.7 mg/L	<5 mg/L
Creatinine (µmol/L)	87 µmol/L	79 µmol/L	91 µmol/L	60-110 µmol/L
Urea (mmol/L)	5.4 mmol/L	2.8 mmol/L	3.3 mmol/L	2.5-7.1 mmol/L
Urine nitrite	Negative	Negative	Negative	Negative
Urine RBCs	Negative	Negative	Negative	Negative
Urine leukocytes	Negative	Negative	Negative	Negative

Case 2

**Figure 2:**
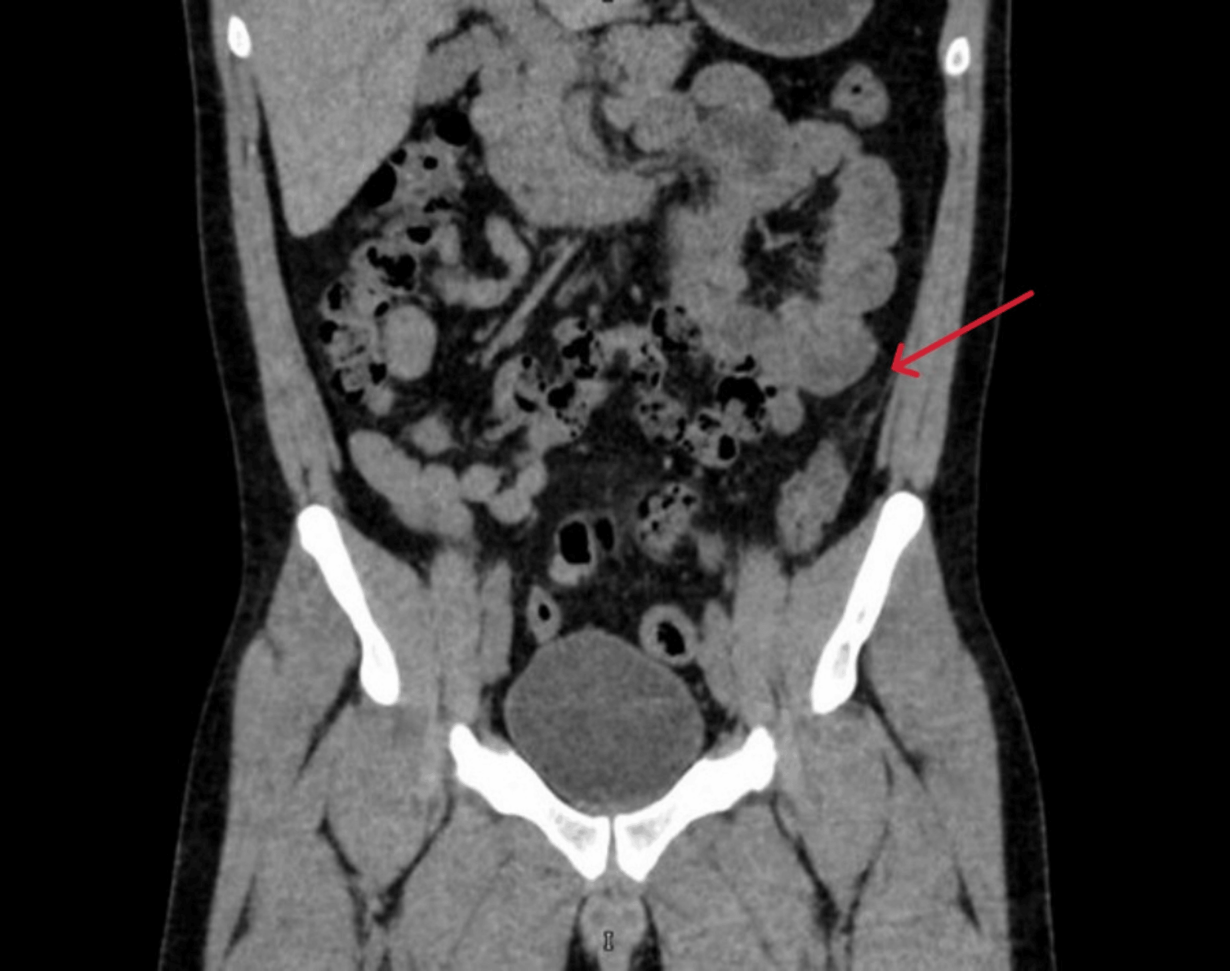
Coronal computed tomography image of cecal epiploic appendagitis The coronal computed tomography image shows a well-defined ovoid fat-density lesion with surrounding inflammatory fat stranding, measuring approximately 2.5 cm along the anterior wall of the cecum (red arrow), consistent with epiploic appendagitis.

A 37-year-old male laborer with a history of cholecystectomy and no chronic illnesses was referred from a health center as a suspected case of acute appendicitis. He presented with a two-day history of abdominal pain that began in the periumbilical region and radiated to the right lower quadrant, associated with nausea and anorexia. He reported a self-limited episode two years ago. On examination, he was alert, well-hydrated, and hemodynamically stable with a temperature of 36.6°C, heart rate (HR) of 78 bpm, and blood pressure (BP) of 139/81 mmHg. Abdominal examination revealed mild guarding, deep tenderness, and mild rebound in the right lower quadrant, without signs of peritonitis. Laboratory investigations showed WBC of 12.56 × 10^9^/L and CRP of 11 mg/L. Other parameters, including renal function, were within normal limits, and urinalysis showed negative nitrites and no RBCs (Table 1). A contrast-enhanced CT scan of the abdomen ruled out appendicitis and demonstrated an ovoid fat-density lesion with surrounding stranding measuring 2.5 cm along the anterior wall of the cecum, consistent with epiploic appendagitis. The appendix was normal, with no cecal wall thickening or diverticulitis (Figure 2). The patient was managed conservatively with paracetamol, fluids, and rest, without antibiotics or surgery. Symptoms resolved within 10 days, and the patient was doing well on follow-up.

Case 3

**Figure 3:**
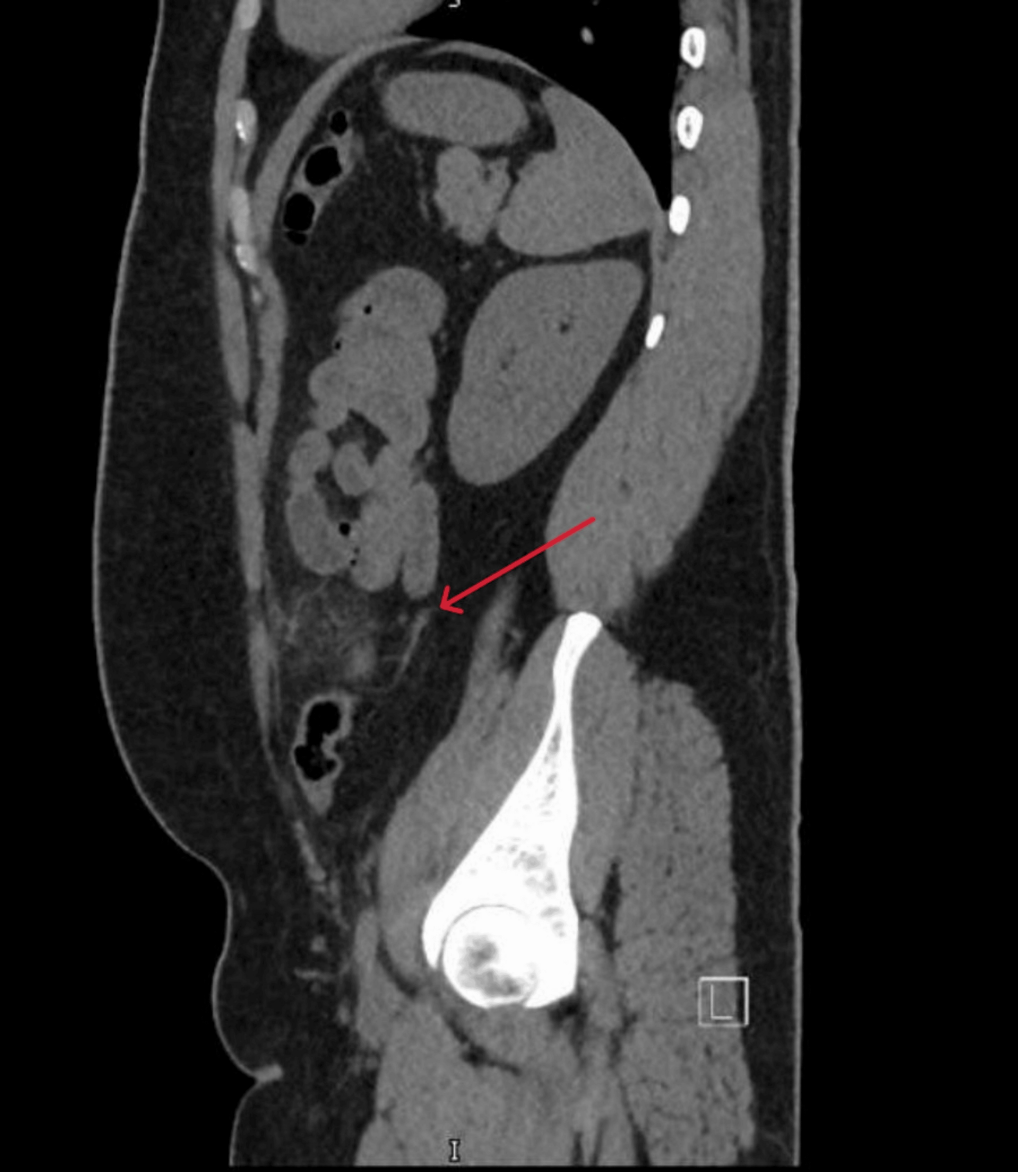
Sagittal computed tomography image of descending colon epiploic appendagitis The sagittal computed tomography image demonstrates an ovoid fat-density lesion adjacent to the lower descending colon, surrounded by inflammatory fat stranding (red arrow), consistent with epiploic appendagitis.

A 37-year-old male patient, previously healthy with no comorbidities, presented with a one-day history of left flank pain and vomiting, associated with dysuria. He denied fever or hematuria. On examination, he was alert, oriented, and hemodynamically stable. Cardiovascular and respiratory examinations were unremarkable, with normal heart sounds and equal bilateral air entry. Abdominal examination revealed a soft, lax abdomen without guarding or rigidity; bowel sounds were present, and tenderness was noted over the left flank. Neurological examination was normal. Laboratory investigations showed a WBC count of 10.2 × 10^9^/L, creatinine of 91 µmol/L, urea of 3.3 mmol/L, and CRP of 11.7 mg/L, and urinalysis was negative for nitrites, RBCs, and pus cells. A contrast-enhanced abdominal CT scan demonstrated an ovoid fat-density lesion adjacent to the lower descending colon, surrounded by inflammatory fat stranding, consistent with epiploic appendagitis. There was no colonic wall thickening, hydronephrosis, or ureteric calculus (Figure 3). The patient was managed conservatively with paracetamol, IV fluids, and rest, without antibiotics or surgery. Symptoms resolved within eight days, and the patient was doing well on follow-up.

The patient was managed conservatively with intravenous fluids and analgesics and was discharged in stable condition. Laboratory findings for all three patients are summarized in Table 1.

## Discussion

In our case series, three male patients, aged between 25 and 37 years, presented with acute localized abdominal pain in different quadrants, mimicking more common diagnoses, such as diverticulitis, appendicitis, or renal colic. All patients were hemodynamically stable and afebrile and had non-specific laboratory findings, with CRP mildly elevated in two cases. Diagnosis was made based on characteristic CT findings of fat-density lesions with surrounding inflammatory changes adjacent to the colon. All patients were managed conservatively with favorable outcomes and no need for surgical intervention. This highlights the importance of clinical suspicion and appropriate imaging in avoiding unnecessary interventions, especially in patients with localized pain and relatively benign systemic signs.

EA is an uncommon and often overlooked cause of acute abdominal pain, typically affecting individuals between the second and fifth decades of life [1,5]. It is characterized by inflammation of the epiploic appendages - fat-filled, serosa-lined pouches that project from the colonic surface [2]. These structures are normally asymptomatic, but torsion or spontaneous venous thrombosis can result in ischemia and localized inflammation. Clinically, EA presents with sudden-onset, localized lower abdominal pain, most commonly in the left lower quadrant, and is often mistaken for more common conditions, such as acute diverticulitis or appendicitis [3,6].

Although the exact incidence remains uncertain, EA is estimated to account for approximately 2-7% of cases initially suspected to be diverticulitis and 0.3-1% of cases thought to be appendicitis [4]. It appears more frequently in males and in individuals with obesity, possibly due to the larger and more mobile nature of epiploic appendages in these populations. Patients typically present with dull, localized, non-radiating pain. Associated symptoms may include mild gastrointestinal complaints, such as nausea, diarrhea, bloating, and early satiety, though systemic symptoms such as fever are usually absent [6]. On examination, most patients are afebrile and hemodynamically stable, with localized tenderness but without signs of peritonitis. A palpable mass may occasionally be present [7].

Distinguishing EA from other acute abdominal pathologies is essential, as misdiagnosis may lead to unnecessary hospital admissions, antibiotic therapy, or surgical intervention. Unlike diverticulitis or appendicitis, patients with EA often appear clinically well and rarely exhibit systemic signs of infection or peritoneal irritation [8]. Contrast-enhanced CT of the abdomen is the gold standard for diagnosing EA. Characteristic findings include a small, oval, fat-density lesion adjacent to the colon, often surrounded by inflammatory fat stranding. While abdominal ultrasound can be useful in certain cases - especially in thin patients or when CT is unavailable - it is operator-dependent and less sensitive. Laboratory findings are generally non-specific; WBC and CRP are often normal or mildly elevated [9].

Management is typically conservative, consisting of non-steroidal anti-inflammatory drugs for symptom control. Most patients experience resolution of symptoms within one to two weeks [2,10]. Antibiotics and hospitalization are rarely necessary unless complications arise. Surgical intervention is reserved for cases with diagnostic uncertainty, failed conservative treatment, or development of complications such as abscess or bowel obstruction [11,12]. Accurate diagnosis not only prevents unnecessary treatments and invasive procedures but also reduces healthcare costs and patient anxiety [2].

The prognosis of EA is excellent, with most patients recovering fully without recurrence or long-term complications. In rare cases, chronic inflammation can lead to fibrosis, calcification, or adhesions, potentially mimicking other intra-abdominal pathologies on future imaging studies. Therefore, clinical awareness and recognition of EA imaging features are critical to avoid misdiagnosis and overtreatment [2].

## Conclusions

This case series highlights epiploic appendagitis as a benign, self-limiting condition that can mimic surgical emergencies. Timely CT imaging allows accurate diagnosis and prevents unnecessary interventions. All patients improved with conservative treatment using paracetamol with IV fluid hydration, without the need for antibiotics or surgery. Increased clinical awareness can reduce misdiagnosis, resource use, and avoidable morbidity. Further research may clarify long-term outcomes and recurrence risks.
